# A Cross Sectional Analysis of Gonococcal and Chlamydial Infections among Men-Who-Have-Sex-with-Men in Cape Town, South Africa

**DOI:** 10.1371/journal.pone.0138315

**Published:** 2015-09-29

**Authors:** Kevin Rebe, David Lewis, Landon Myer, Glenn de Swardt, Helen Struthers, Monika Kamkuemah, James McIntyre

**Affiliations:** 1 Anova Health Institute, Johannesburg and Cape Town, South Africa; 2 University of Cape Town, Division of Infectious Diseases and HIV Medicine, Department of Medicine, Cape Town, South Africa; 3 Centre for HIV and Sexually Transmitted Infections, National Institute for Communicable Diseases, National Health Laboratory Service, Johannesburg, South Africa; 4 Division of Medical Microbiology, University of Cape Town, Cape Town, South Africa; 5 Western Sydney Sexual Health Centre, Parramatta, Australia; 6 Centre for Infectious Diseases and Microbiology & Marie Bashir Institute for Infectious Diseases and Biosecurity, Westmead Clinical School, University of Sydney, Sydney, Australia; 7 Division of Epidemiology & Biostatistics, School of Public & Family Medicine, University of Cape Town, Cape Town, South Africa; Emory University Rollins School of Public Health, UNITED STATES

## Abstract

**Background:**

Men-who-have-sex-with-men (MSM) are at high risk of HIV and sexually transmitted infection (STI) transmission. Asymptomatic STIs are common in MSM and remain undiagnosed and untreated where syndromic management is advocated. Untreated STIs could be contributing to high HIV rates. This study investigated symptomatic (SSTI) and asymptomatic STIs (ASTIs) in MSM in Cape Town.

**Methods:**

MSM, 18 years and above, were enrolled into this study. Participants underwent clinical and microbiological screening for STIs. Urine, oro-pharyngeal and anal swab specimens were collected for STI analysis, and blood for HIV and syphilis screening. A psychosocial and sexual questionnaire was completed. STI specimens were analysed for *Neisseria gonorrhoeae* (NG) and *Chlamydia trachomatis* (CT) infection.

**Results:**

200 MSM were recruited with a median age of 32 years (IQR 26–39.5). Their median number of sex partners within the last year was 5 (IQR 2–20). 155/200 (78%) reported only male sex partners while 45/200 (23%) reported sex with men and women. 77/200 (39%) reported transactional sex. At enrolment, 88/200 (44%) were HIV positive and 8/112 (7%) initially HIV-negative participants seroconverted during the study. Overall, 47/200 (24%) screened positive for either NG or CT. There were 32 MSM (16%) infected with NG and 7 (3.5%) of these men had NG infections at two anatomical sites (39 NG positive results in total). Likewise, there were 23 MSM (12%) infected with CT and all these men had infections at only one site. Eight of the 47 men (17%) were infected with both NG and CT. ASTI was more common than SSTI irrespective of anatomical site, 38 /200 (19%) versus 9/200 (5%) respectively (p<0.001). The anus was most commonly affected, followed by the oro-pharynx and then urethra. Asymptomatic infection was associated with transgender identity (OR 4.09 CI 1.60–5.62), ≥5 male sex partners in the last year (OR 2.50 CI 1.16–5.62) and transactional sex (OR 2.33 CI 1.13–4.79) but not with HIV infection.

**Conclusions:**

Asymptomatic STI was common and would not have been detected using a syndromic management approach. Although molecular screening for NG/CT is costly, in our study only four MSM needed to be screened to detect one case. This supports dual NG/CT molecular screening for MSM, which, in the case of confirmed NG infections, may trigger further culture-based investigations to determine gonococcal antimicrobial susceptibility in the current era of multi-drug resistant gonorrhoea.

## Introduction

Men-who-have-sex-with-men (MSM) are at high risk of acquiring and transmitting HIV and other sexually transmitted infections (STIs) and MSM have been identified as a key population requiring targeted HIV prevention interventions. [1–5] The prevalence of HIV among South African MSM is high with an estimated range of 10–43%. [6–9] Reasons for this are complex and include high biological risk of HIV transmission during unprotected receptive penile-anal sex (compared to penile-vaginal sex) in addition to behavioural and sexual network factors. [10,11] Furthermore, the presence of prior STIs is associated with increased HIV vulnerability among MSM and treating STIs is believed to be a valid strategy for decreasing HIV transmission. [2]

Anatomical sites of sexual infection in MSM may differ from those in men who have sex exclusively with women, and include oro-pharyngeal and anal infections. STIs at these sites may be symptomatic (SSTI) or asymptomatic (ASTI) and asymptomatic disease is more likely to be inadequately diagnosed and treated. [12,13] South Africa obligates the use of syndromic management of STIs for all patients utilizing the public sector health system. Syndromic management is a tool for managing symptomatic STIs and inherently does not allow for diagnosis of ASTI and therefore may not be responsive to the health needs of MSM. [14,15] In addition, South African STI guidelines do not include guidance on how to manage ano-rectal discharge as a syndrome.

Due to the high incidence of ASTIs among MSM globally, the World Health Organization has produced guidelines advocating for empiric STI treatment in asymptomatic high-risk MSM. [2,15] Few data are available for South Africa and indeed for most African countries to allow for the assessment of the suitability of this recommendation. A study in 43 MSM in Kenya reported that 11 (26%) screened positive for either *Neisseria gonorrhoeae* (NG) or *Chlamydia trachomatis* (CT) infection. Only 2 out of 43 participants reported symptoms, i.e. the majority of infections were asymptomatic and these patients would not have been treated unless screening had taken place. However, they would have benefited from empiric ASTI treatment according to the WHO guidance. [13]

## Rationale and Aims

Screening for both SSTIs and ASTIs in MSM has become standard of care in many developed world nations but does not occur in the state healthcare sector in South Africa and many developing nations. [2,15,16] The burden of STIs and ASTIs in South African MSM remains unknown and, due to the lack of clinical services with high numbers of MSM attendees, no systematic surveillance is occurring. [14] Similarly, the contribution of sexual infections to high HIV rates among local MSM remains poorly understood and described.

This study aimed to describe the burden of symptomatic and asymptomatic NG/CT infection in MSM attending the Ivan Toms Centre for Men’s Health (ITCMH). Secondary aims included investigation of risk factors associated with molecular detection of NG/CT, including the effect of HIV sero-status, and the number of MSM needing STI screening to detect one ASTI case.

## Methods

### Study Setting

State-sector MSM-targeted sexual health services have existed in South Africa since 2009 when the Anova Health Institute’s Health4Men programme was launched as a collaborative project with the Department of Health with support and funding from PEPFAR/USAID. [17] The longest running of these services is the Ivan Toms Centre for Men’s Health (ITCMH) in Cape Town which functions as a centre of excellence for developing and delivering locally-appropriate MSM sexual health care. This clinic has provided services to more than 6,500 MSM to date. [18,19] The clinic is a primary-level, sexual health and wellness service focusing on HIV and STI diagnosis, treatment and prevention and includes biomedical, psychosocial, educational and community components. The ITCMH is situated alongside major car, taxi and train commuter routes and attracts MSM clients with a range of sexual identities from a wide geographic catchment area surrounding Cape Town. Since the clinic attracts clients from such a diverse spectrum of MSM in different geographic areas, and with diverse socio-economic status and cultural backgrounds, it is likely that MSM attending the clinic are broadly representative of urban and peri-urban MSM in and around the City of Cape Town.

### Recruitment

All MSM clients attending the clinic between January-July 2012 were screened for enrolment irrespective of their reason for attendance. Only two potential study participants declined enrolment after screening. A sample of 200 consecutive MSM attending the ITCMH in Cape Town was enrolled. The importance of screening for and treating both symptomatic and asymptomatic STIs was promoted at community level by Health4Men outreach officers, events in public spaces frequented by MSM and by staff at the ITCMH.

Inclusion criteria were: over 18 years of age, sex (insertive or receptive, oral or anal sex or anilingus) with another man within the previous year and ability to understand the study and provide informed consent to participate. MSM were excluded if they were taking treatment for a recent STI or had taken such treatment in the past two weeks prior to study screening, or were unavailable to attend the clinic for a follow-up study visit. Ethical approval for this study was obtained from the University of Cape Town Human Sciences Research Ethics Committee. (Protocol number: HSREC 419/2011.) All study participants provided written informed consent prior to study enrolment. The study complies with the Helsinki Declaration and all principles of Good Clinical Practice.

### Study Activities

Enrolled participants attended two study-related visits two weeks apart. At the first visit, participants completed researcher-administered interviews. Symptoms of urethral, anal and oro-pharyngeal pain, discharge, itch or irritation were assessed. A clinical examination to identify STIs and HIV-related clinical conditions was performed by a qualified clinician with experience in providing health care to MSM. Participants provided self-collected urine and clinicians collected rectal and oro-pharyngeal swabs. Phlebotomy was performed for syphilis and HIV testing where status was unknown or uncertain. HIV positive participants had a CD4 count performed. All newly diagnosed HIV positive participants received appropriate HIV counselling and linkage to care.

At the second study visit, participants were informed of their specimen results and diagnosed infections were treated with appropriate antibiotics as per in-country guidelines. [15] Gonorrhoea was treated with single-dose cefixime 400mg orally and chlamydial infections were treated with doxycycline 100mg orally 12 hourly for seven days. Sexual contacts were traced for treatment and where necessary, patients were linked back to the ITCMH for relevant chronic care. At both study visits, participants received sexual risk-reduction counselling, condoms and latex-compatible sexual lubricants and a range of information and educational materials.

### Laboratory Analysis

Urine, oro-pharyngeal and anal swabs were analysed for NG/CT infection using the RNA-based Aptima Combo 2 assay (Hologic-Gen-Probe, San Diego, CA, USA). Syphilis analysis was performed using rapid kit SD Bioline syphilis 3.0 test (Standard Diagnostics, Gyeonggi-do, Republic of Korea). Positive results were confirmed using the Immunotrep Carbon Antigen rapid plasma reagin (RPR) and the Immunotrep *Treponema pallidum* haemagglutination assay (Omega Diagnostics, Alva, United Kingdom). HIV testing utilized serial rapid kit ELISA methodology. The SD Bioline HIV 1/2 3.0 test kit (Standard Diagnostics, Gyeonggi-do, Republic of Korea) was used for initial screening and positive results were confirmed with the Determine HIV-1/2 kit (Alere Medical, Matsuhidai, Japan). Positive results on both rapid kits were considered to be truly HIV positive. A single negative kit result was regarded as a true negative but study participants were counselled to undergo post-window period re-testing. Pan-leucogated CD4 analysis was performed for all HIV positive participants (Beckman Coulter, Brea, CA, USA).

### Data Analysis

Data were analysed using Stata 12.0 (Stata Corporation, College Station, TX, USA). Descriptive statistics (proportions, means with SD, medians with IQR) were used to summarize the data. Bivariate analysis employed chi-squared and Fishers exact tests (for comparisons of proportions) and Kruskall-Wallis tests (for comparisons of medians).

## Results

200 MSM were enrolled during the recruitment period. Their median age was 32 years (IQR 26–39.5 years). All participants lived locally in Cape Town or its surrounds, with a significantly greater proportion of black participants, 40/200 (65%), living in peri-urban areas compared to the other racial groups. Most (73%) of participants had completed high school, 42% had a tertiary level qualification and 68% were employed in full or part-time work. A total of 83/200 (42%) of participants were circumcised. Of these, 56 (28%) were medical circumcisions and 27 (14%) were traditional circumcisions. Baseline characteristics of the participants by population group and the range of sexual activities and risk behaviours are summarized in Table 1.

**Table 1:** Baseline Characteristics, Sexual Behaviour and STI Risks Stratified by Population Group.

Variable	All Patients (N = 200)	Black 62 (31%)	White 71 (36%)	Coloured /Other 67 (33%)	P value
**Baseline characteristics stratified by population group**					
Age [years (IQR)]	32 (26–39.5)	29 (24–35)	34 (27–44)	32 (26–39)	0.001
Gender					
Male	185 (93%)	55 (89%)	71 (100%)	59 (88%)	
Transgender women	15 (8%)	7 (11%)	0	8 (12%)	0.011
Education					
Completed high school	145 (73%)	45 (73%)	63 (89%)	37 (55%)	<0.001
Tertiary qualification	84 (42%)	22 (35%)	46 (65%)	16 (24%)	<0.001
Residence					
City or suburbs	147 (73.5%)	22 (35%)	64 (90%)	61 (91%)	
Peri-urban	53 (26.5%)	40 (65%)	7 (10%)	6 (9%)	<0.001
Employed	137 (68%)	39 (63%)	54 (76%)	44 (66%)	0.220
Cell phone	159 (79.5%)	52 (84%)	68 (96%)	39 (58%)	<0.001
Internet Access	151 (75.5%)	44 (71%)	68 (96%)	39 (58%)	<0.001
Total circumcised	83 (42%)	33 (53%)	28 (39%)	22 (33%)	0.058
**Sexual behavior characteristics in the past 12 months (last 10 sex partners) and other risk factors for STIs stratified by population group**					
**Sex with**					
Men only	155 (78%)	48 (77%)	65 (92%)	42 (63%)	
Men and women	43 (22%)	14 (23%)	6 (8%)	25 (37%)	<0.001
**Number of male sex partners (median and IQR)**	5 (2–20)	5 (2–12)	5 (3–20)	6 (2–24)	0.803
<5 male sex partners	88 (44%)	27 (44%)	33 (46%)	28 (42%)	
≥5 male sex partners	112 (56%)	35 (56%)	38 (54%)	39 (58%)	0.854
**Group sex in the past year**	59 (30%)	16 (26%)	27 (38%)	16 (24%)	0.142
**Sex under influence of alcohol (ever)**	137 (69%)	45 (73%)	49 (69%)	43 (64%)	0.587
**Sex under influence of drugs (ever)**	105 (53%)	14 (23%)	45 (63%)	46 (69%)	<0.001
**Crystal methamphetamine use (ever)**	73 (37%)	12 (19%)	23 (32%)	38 (57%)	<0.001
**Any transactional sex in past year**	77 (38.5%)	22 (35%)	16 (23%)	39 (58%)	<0.001
None	123 (62%)	40 (64%)	55 (77%)	28 (42%)	
Paid for sex	24 (12%)	6 (10%)	11 (16%)	7 (10%)	
Received payment for sex	36 (18%)	13 (21%)	3 (4%)	20 (30%)	
Paid for and received payment for sex	17 (8%)	3 (5%)	2 (3%)	12 (18%)	<0.001
**STI-diagnosis in the past year**	47 (23%)	20 (32%)	13 (18%)	14 (21%)	0.138
**HIV test in past year**					
No (or don’t know result)	93 (46.5%)	34 (55%)	30 (42%)	29 (43%)	
Yes	107 (53.50%)	28 (45%)	41 (58%)	38 (57%)	0.283
**Type of Sex in the last 12 months** *					
Insertive anal sex	143 (72%)	37 (60%)	61 (86%)	45 (67%)	0.002
Receptive anal sex	140 (70%)	47 (76%)	55 (77%)	38 (57%)	0.014
Both (insertive and receptive) anal sex	92 (46%)	24 (39%)	49 (69%)	19 (28%)	<0.001
Insertive oral sex	163 (82%)	39 (63%)	67 (94%)	57 (85%)	<0.001
Receptive oral sex	174 (87%)	46 (74%)	69 (97%)	59 (88%)	<0.001
Both (Insertive and receptive) oral sex	149 (75%)	33 (53%)	66 (93%)	50 (75%)	0.023
Performed analingus (rimming)	77 (39%)	18 (29%)	38 (54%)	21 (31%)	0.005
Received rimming	96 (48%)	25 (40%)	38 (54%)	33 (49%)	0.305
Both performed and received rimming	62 (31%)	16 (26%)	27 (38%)	19 (28%)	0.116

* Participants may have reported engaging in more than one of the sexual activity categories listed in this table.

More than half the participants (52%) reported having a boyfriend whom we defined as the primary sexual partner. The median number of sex partners in the year prior to recruitment was 5 (IQR 2–20). One hundred and fifty-five participants (78%) reported sex with men only and 45/200 (23%) reported sex with men and women. Any transactional sex was reported by 77/200 (38%); 12% reporting having paid for sex, 18% received payment for sex and 8% paid for and received payment for sex.

Fifty seven (29%) reported clinical symptoms suggestive of an STI: 10(5%) had a urethral discharge, 26(13%) reported oro-pharyngeal pain and 22 (11%) reported anal discharge or pain. Overall, 47 participants (23%) had a previous diagnosis of an STI in the 12 months prior to enrolment and 21 (10%) had been previously been treated for syphilis.

**Fig 1:**
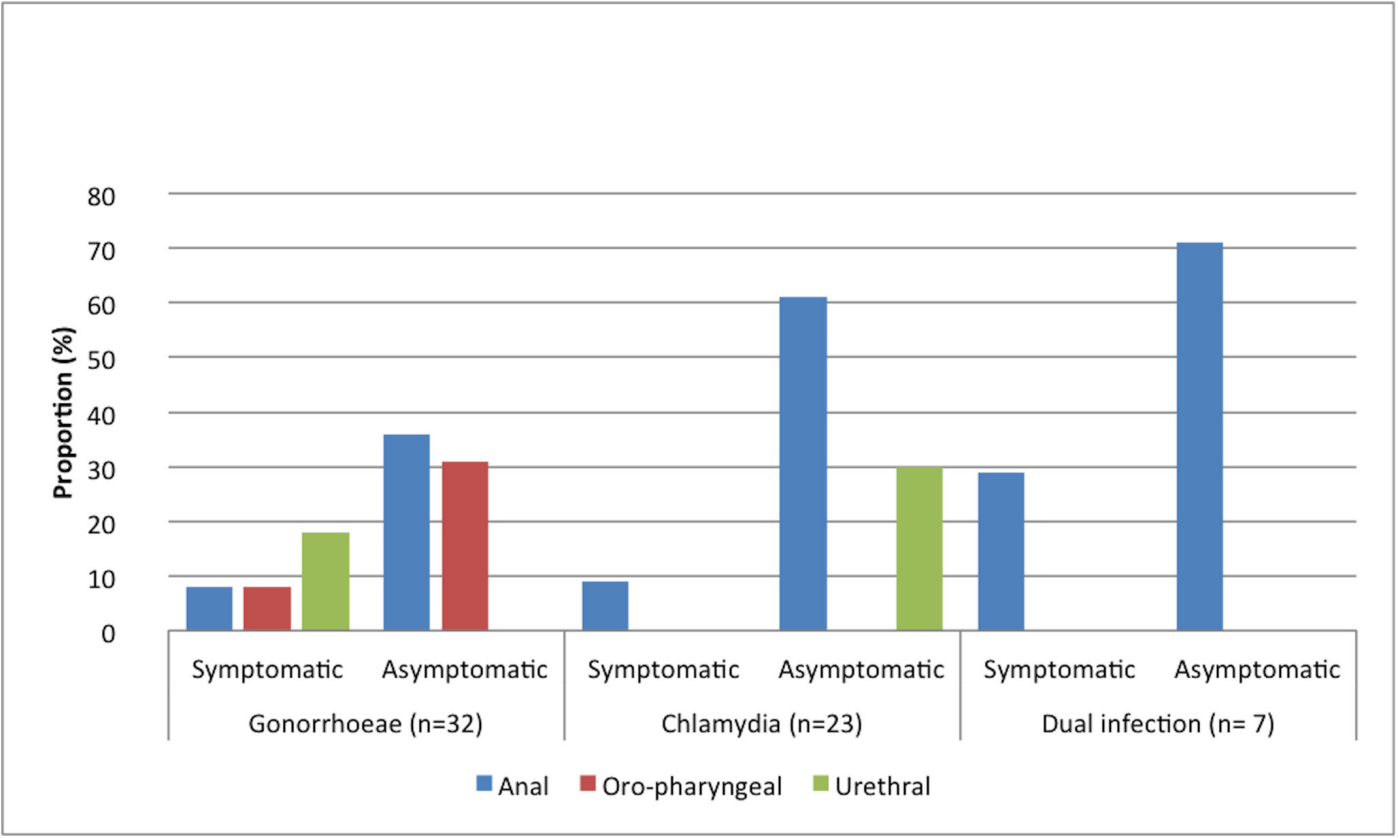
Symptomatic and asymptomatic gonorrhoea and chlamydia by site of infection.

STI results are summarised in Fig 1 and Table 2.

**Table 2:** Symptomatic and asymptomatic Gonorrhoea and Chlamydia by site of infection.

	*N*. *gonorrhoeae* site-related infections in 32 MSM^1^		*C*. *trachomatis* site-related infections in 23 MSM		Dual infections at the same site (n = 7^2^)	
	Symptomatic	Asymptomatic	Symptomatic	Asymptomatic	Symptomatic	Asymptomatic
Anal	3 (8%)	14 (36%)	2 (9%)	14 (61%)	2 (29%)	5 (71%)
Oro-pharyngeal	3 (8%)	12 (31%)	0	0	0	0
Urethral	7 (18%)	0	0	7 (30%)	0	0
**Any**	**13 (33%)**	**26 (67%)**	**2 (9%)**	**21 (91%)**	**2 (29%)**	**5 (71%)**
Total MSM with symptomatic NG/CT infections: 9/200 (5%)						
Total MSM with asymptomatic NG/CT infections: 38/200 (19%)						
Total proportion of ASTIs were significantly more common than total proportion of SSTIs, p <0.001						

^1^ Seven MSM had dual *N*. *gonorrhoeae* and *C*. *trachomatis* infections at the same anatomical site

^2^ There was one MSM with dual infection at different anatomical sites (NG positive in oro-pharynx and CT positive in urine)

Overall, 47/200 (24%) screened positive for either NG or CT. There were 32 MSM (16%) infected with NG and 7 of these men had NG infections at two anatomical sites (39 NG positive results in total). Likewise, there were 23 MSM (12%) infected with CT and all these men had infections at only one site. Eight of the 47 men (17%) were infected with both NG and CT.

Nine of 200 (5%) of study participants with these infections were symptomatic whilst 38/200 (19%) were asymptomatic despite testing positive for either NG or CT or both. Gonorrhoea was more likely to be asymptomatic in the pharynx or anus compared to the urethra (P<0.05) but no such difference was seen for chlamydial infections since all urethral chlamydia infections in this study were asymptomatic. Of those who screened negative for NG or CT, 48/153 (31%) reported symptoms that would have incorrectly resulted in empiric STI management if empiric syndromic guidelines were used.

Eighty-eight (44%) participants had prevalent HIV at enrolment and 8/112 (7%) of initially HIV negative participants were newly diagnosed with HIV during the study. The mean CD4 count of HIV positive MSM was 420 cells/mm^3^ (SD 176.8 cells/ mm^3^) and 46/88 (52%) were receiving antiretroviral therapy (ART). No statistically significant difference in SSTI or ASTI by HIV status was found, except for syphilis, which was more common among HIV positive participants. Refer Table 3.

**Table 3:** Sexually transmitted infections by HIV sero-status.

Variable n (%)	Total (n = 200)	HIV-positive 88 (44%)	HIV-negative 112 (56%)	P.value
**Active Syphilis (TPHA/rapid and RPR Positive)**	22 (11%)	18 (20%)	4 (4%)	<0.001
Syphilis (RPR Positive)	23 (12%)	19 (22%)	4 (4%)	<0.001
Syphilis (TPHA Positive)	21 (11%)	17 (19%)	4 (4%)	<0.001
**Chlamydial Infection**				
Urethral	7 (3.5%)	0 (0%)	7 (6%)	0.017
Anal	16 (8%)	8 (9%)	8 (7%)	0.614
Any site	23 (11.5%)	8 (9%)	15 (13%)	0.344
**Gonorrhoea**				
Urethral	7 (3.5%)	2 (2%)	5 (4%)	0.403
Oro-pharyngeal	15 (8%)	7 (8%)	8 (7%)	0.829
Anal	17 (8%)	11 (13%)	6 (5%)	0.072
Any site	32 (16%)	17 (19%)	15 (13%)	0.257
**Chlamydial & Gonorrhoea Co-infection**	8 (4%)	5 (6%)	3 (3%)	0.282
**Any NGCT**	47 (23.5%)	20 (23%)	27 (24%)	0.819
Symptomatic and NG/CT positive	9/47 (19%)	4 (20%)	5 (19%)	0.898
Asymptomatic and NG/CT positive	38/47 (81%)	16 (80%)	22 (81%)	
**All STI**	60 (30%)	31 (35%)	29 (26%)	0.153
**Clinical Diagnosis**				
Urethritis	11 (5.5%)	3 (3%)	8 (7%)	0.250
Pharyngitis	8 (4%)	2 (2%)	6 (5%)	0.269
Proctitis	5 (2.5%)	3 (3%)	2 (2%)	0.465
Other	85 (42.5%)	46 (52%)	39 (35%)	
**STI diagnosis in the past 12 months**				
Overall	47 (23.5%)	28 (32%)	19 (17%)	0.014
Urethral discharge	16/47 (34%)	7/28 (25%)	9/19 (47%)	0.112
Genital herpes	3/47 (6%)	1/28 (4%)	2/19 (11%)	0.338
Syphilis	21/47 (45%)	16/28 (57%)	5/19 (26%)	0.037
Anal HPV	4/47 (9%)	2/28 (7%)	2/19 (11%)	0.544

In further bivariate analysis, ASTI was strongly associated with identifying as transgender as opposed to MSM or gay (OR = 4.09, CI 1.38–12.12), having five or more male sex partners within the past 12 months compared to fewer than 5 partners (OR = 2.56, CI 1.16–5.62) and engaging in transactional sex in the past year compared to reporting no transactional sex (OR = 2.33, CI 1.13–4.79). Having insertive oral sex, compared to those reporting no insertive oral sex, appeared to be protective (OR = 0.38, CI 0.17–0.87) but when compared to receptive oral sex, there was no significant protective effect (OR = 0.52, CI 0.16–1.64) but this was likely due to the small sample size. Refer Table 4.

**Table 4:** Factors associated with asymptomatic STIs (Gonorrhoea and Chlamydia).

Variable	ASTI *Crude OR (95% CI)*
Age (years)	0.97 (0.93–1.01)
Gender	
Male	1.00
Transgender women	4.09 (1.38–12.12)
Completed high school (versus not)	1.06 (0.47–2.37)
Residence	
City or suburbs	1.00
Peri-urban	0.85 (0.37–1.94)
Employed (versus not)	0.48 (0.23–1.00)
Male Circumcision (MC)	
Medical MC or Traditional MC	1.00
No	1.78 (0.84–3.78)
Sex Partners	
Men only	1.00
Men and women	0.61 (0.24–1.57)
Number of male sex partners	
<5 male sex partners	1.00
≥5 male sex partners	2.56 (1.16–5.62)
Sex under influence of alcohol. Ever (versus never)	0.85 (0.40–1.81)
Sex under influence of drugs. Ever (versus never)	1.59 (0.77–3.29)
Crystal Methamphetamine use	1.8 (0.88–3.70)
Transactional Sex in past year (versus not)	2.33 (1.13–4.79)
STI-diagnosis in the past year (versus not)	1.97(0.89–4.37)
HIV positive (versus negative)	0.91 (0.44–1.87)
Type of Sex in the last 12 months	
Insertive anal sex	0.79 (0.37–1.72)
Receptive anal sex	1.28 (0.58–2.85)
Insertive oral sex	0.38 (0.17–0.87)
Receptive oral sex	0.88 (0.30–2.54)
Performed rimming	0.88 (0.42–1.83)
Received rimming	0.94 (0.46–1.91)

In a separate analysis, risk factors for any STI (SSTI and ASTI) included having had an STI diagnosis in the past year (OR = 3.00, CI 1.52–5.94), having had sex under the influence of drugs in the last six months (OR = 2.12, CI 1.11–4.07), having had sex with an internet-sought partner in the past year (OR = 1.98, CI 1.07–3.66) and having had sex at a sex-on-site venue in the past year (OR = 1.92, CI 1.02–3.64). Logistic regression modelling suggested that these associations were not significantly confounded by age (data not shown).

## Discussion

This study is the first to investigate asymptomatic NG/CT infections among MSM in South Africa. A high burden of NG/CT infection (24% screened positive for NG/CT at any site) was demonstrated in our participants, most of whom were asymptomatic. This is similar to rates found in other African developing nations. A study from coastal Kenya reported that 11/43 (26%) (95% CI 14–41%) screened positive for NG or CT or both. [13] A later study from the same researchers documented a 3 month incidence of any rectal NG/CT asymptomatic infection in men reporting unprotected receptive anal sex of 39% (95% CI 24–65%).[20] A study investigating rectal STIs among a sample of 172 MSM in two cities in Tanzania reported rates of curable STIS (NG/CT and syphilis) as 21% (large metropolis) and 4.4% (provincial city). [21] Similar rates of asymptomatic infection have been reported in the USA: 24% NG or CT infections among a cohort of 99 HIV positive US Marine Corps men, and 38% with rectal NG or CT infections in 326 MSM attending an STI clinic in the US Midwest. [22,23]

Results from our study demonstrate risky sexual behaviours among local MSM. Enrolled participants had a median of five partners within the past year, condom use was low, transactional sex was common and almost half the participants were HIV positive. Almost a quarter had been diagnosed and treated for an STI in the past 12 months. These data demonstrate the need for effective HIV and STI prevention interventions that are MSM-focused.

High rates of asymptomatic NG/CT are of particular relevance to resource-limited settings where STIs are managed syndromically and most asymptomatic STIs remain untreated due to lack of laboratory-based screening policies, even in key populations at high risk of STI acquisition, such as MSM. The inability to diagnose asymptomatic STIs in MSM leads to under-treatment, poor STI control and possibly drives HIV transmission in this high HIV-risk population. The relationship between STIs and HIV is a complex one which has been examined in many studies. It is biologically plausible that STIs are a driver of HIV transmission as they cause gaps in mucosal integrity as well as promoting inflammation which drives HIV replication. [24,25,26] Studies have demonstrated a link between urethral STIs and increases in HIV viral load, although there have also been negative studies in this regard. [24,27] A US-based study modelled the effect of STIs on HIV rates among young MSM in Chicago and reported that approximately 15% of new HIV infections in this model were attributable to NG/CT infections. [28] The proportion of HIV infections attributable to NG/CT has not been modelled for South African MSM, however data are available to show this relationship in cohorts of South African women. [29] No formal meta-analysis has been conducted to answer these questions among MSM, although the World Health Organisation has assessed relevant data and has recommended that screening and treatment of STIs should form part of the core package or HIV risk reduction among MSM.

Additionally, the correlation between symptoms and microbiologically confirmed STIs was poor in our study which emphasizes the well-recognized over-treatment associated with the syndromic management approach. Although most NG/CT cases were diagnosed in asymptomatic MSM, the majority of asymptomatic MSM were not infected with either pathogen. Therefore, following the WHO recommendations for empiric treatment of STIs among high risk asymptomatic MSM would also result in substantial over-treatment. The degree of overtreatment could be reduced if NG/CT treatment could be targeted at those MSM defined risk factors for NG/CT infections. Our study suggests that MSM could be prioritized for targeted STI screening if they have had (i) an STI diagnosis in the past year, (ii) sex under the influence of drugs in the last six months, (iii) sex with an internet-sought partner in the past year, or (iv) sex at a sex-on-site venue.

The high cost of molecular detection of STIs remains a significant barrier to appropriate STI services in South Africa and other resource-poor nations (approximately $150 per participant to screen three anatomical sites). Cheaper platforms, and ideally rapid point-of-care tests, would be necessary before NG/CT screening could be incorporated into standard practice. Nevertheless, our data demonstrate that only four MSM clients would need to be screened in order to diagnose an STI, making a strong case for directed screening in MSM.

The World Health Organization currently recommends intermittent empiric treatment for asymptomatic STIs among high risk MSM [2]. Given that gonococcal cephalosporin resistance rates are increasing globally, that resistance is more common in MSM compared to heterosexual people and that cases of resistance and treatment failure are now being detected in South African MSM, targeted screening, with subsequent bacteriological culture and antimicrobial susceptibility testing for those MSM diagnosed with gonorrhoea, may be a preferable strategy.[12,14,30] Based on the high incidence of ASTIs demonstrated in this MSM study cohort, we recommend at least sentinel screening of MSM in centres of excellence for MSM health care to determine asymptomatic rates. For countries that are unable to operationalize sentinel or widespread direct screening for financial, logistic or other reasons, following the WHO guideline for empiric STI treatment for high risk MSM remains the best current approach.

Potential weaknesses of our study include the relatively small sample size which limited the statistical power for investigation of associations during study data analysis. Also study participants were recruited from a well-known MSM clinic in metropolitan Cape Town (i.e. a clinic-based sample) and may not be representative of other MSM in South Africa or globally. STIs other than NG/CT are not the focus of this paper and will be reported in future publications. Despite these weaknesses, data from this study provide important insights into the STI disease burden among South African MSM and may assist health planners and implementers to improve targeted programs in an evidence-based manner: something that has been difficult given the general lack of knowledge relating to STIs in African MSM.

## Conclusions

In conclusion, it is important to find the correct balance between cost effective empiric treatment of symptomatic STIs and expensive screening for asymptomatic STIs in key populations at high risk of HIV infection. More research is required in this field to inform health planners how best to design and implement such programs. Also, since STI and HIV rates are likely to vary between populations and geographic settings, local health planners will need to ensure that existing guidance is relevant to their epidemiological setting. Our data support the recommendation of sentinel screening of STIs among MSM for countries that can afford such programs.
